# Serum ferritin level during hospitalization is associated with Brain Fog after COVID-19

**DOI:** 10.1038/s41598-023-40011-0

**Published:** 2023-08-11

**Authors:** Teruyuki Ishikura, Tomohito Nakano, Takaya Kitano, Takechiyo Tokuda, Hisae Sumi-Akamaru, Takashi Naka

**Affiliations:** 1 Department of Neurology, Higashiosaka City Medical Center, 3-4-5 Nishiiwata, Higashiosaka, Osaka 578-8588 Japan; 2 Department of Molecular Neuroscience, Graduate School of Medicine, Osaka University, Suita, Osaka Japan; 3 Department of Neurology, Osaka University Graduate School of Medicine, Suita, Osaka Japan; 4 Krembil Research Institute, Toronto Western Hospital, University Health Network, Toronto, ON Canada

**Keywords:** Medical research, Inflammation

## Abstract

The coronavirus disease 2019 (COVID-19) remains an epidemic worldwide. Most patients suffer residual symptoms, the so-called “Long COVID,” which includes respiratory and neuropsychiatric symptoms. Brain Fog, one of the symptoms of Long COVID, is a major public health issue because it can impair patients’ quality of life even after recovery from the disease. However, neither the pathogenesis nor the treatment of this condition remains unknown. We focused on serum ferritin levels in this study and collected information on the onset of Brain Fog through questionnaires and found that high ferritin levels during hospitalization were associated with the occurrence of Brain Fog. In addition, we excluded confounders as far as possible using propensity score analyses and found that ferritin was independently associated with Brain Fog in most of the models. We conducted phase analysis and evaluated the interaction of each phase with ferritin levels and Brain Fog. We found a positive correlation between serum ferritin levels during hospitalization and Brain Fog after COVID-19. High ferritin levels in patients with Brain Fog may reflect the contribution of chronic inflammation in the development of Brain Fog. This study provides a novel insight into the pathogenic mechanism of Brain Fog after COVID-19.

## Introduction

Severe acute respiratory syndrome coronavirus 2 (SARS-CoV-2) is endemic. Although vaccines reduce the mortality rate^1^, more than 80% of patients reportedly suffer some residual symptoms even several months after onset^2^. Post-coronavirus disease (COVID) syndrome (also termed Long COVID) includes respiratory symptoms such as dyspnea, cough, and phlegm, and symptoms related to neuropsychiatric symptoms such as olfactory and taste disorders, fatigue, cognitive impairment, concentration or memory issues and delirium^3^. The definition of Long COVID varies; the Centers for Disease Control and Prevention (CDC) explains that symptoms begin 4 weeks after infection^4^, and the World Health Organization (WHO) states that symptoms begin 12 weeks after infection and last for at least 2 months^5^. Long COVID is a significant public health issue because it can severely impact work and quality of life, even after a mild COVID infection^6–8^. One of the most common and serious symptoms of Long COVID is Brain Fog. It is defined as an altered state of consciousness in which a person is less wakeful, aware, alert, and focused than usual^9,10^. Brain Fog is not specific to long COVID, and the term Brain Fog was reported before the coronavirus disease 2019 (COVID-19) epidemic and is a general term for being dazed or foggy. Brain Fog has received increased attention after the COVID-19 epidemic. Many people suffer from Brain Fog after COVID-19, which reduces the patient's quality of life and intellectual work. A previous report indicated that Brain Fog was reported in 81% of patients who self-presented to a Neuro-COVID clinic, and some of them showed abnormalities detected on brain MRI^11^.

Brain Fog is thought to be caused by neuroinflammation^12^. Compared to the prevalence of Brain Fog which we empirically know after ordinary upper respiratory viral infections, the prevalence of 17.8% 2 months after COVID-19 infection is quite high^13^. This difference between common respiratory viral infections and COVID-19 shows that neuroinflammation leading to Brain Fog may be related to the specific pathogenesis of COVID-19. Recently, it was reported that the overactivation of brain monocytes leads to neuroinflammation in patients with COVID-19^14,15^.

In this context, we hypothesized that the overactivation of monocytes in COVID-19 causes Brain Fog. Here, we focused on serum ferritin, a common clinical marker of inflammation, and investigated the association between ferritin levels and Brain Fog after COVID-19.

## Methods

### Participants and surveys of residual symptoms

Higashiosaka City Medical Center is the central public hospital of Higashi-Osaka City, a city in the Kinki region of Japan, with a total population of approximately 500,000. This hospital is a tertiary medical center in Osaka Prefecture offering treatment for COVID-19. All patients were diagnosed with COVID-19 by positive nasopharyngeal swab polymerase chain reaction (PCR). In general, patients admitted to our hospital were either elderly or at a high risk of severe respiratory failure; however, patients who required tracheal intubation were transferred to other hospitals that provided intensive care for COVID-19 respiratory failure. After receiving acute medication for COVID-19, they were discharged or transferred to rehabilitation hospitals, long-term care hospitals, or intensive care hospitals for tracheal intubation management for severe respiratory failure.

**Figure 1:**
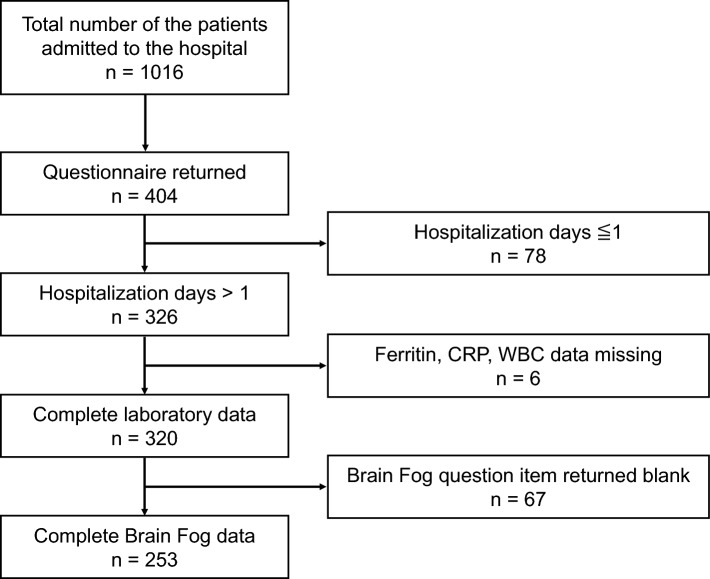
Patient selection.

We surveyed post-COVID syndrome by sending questionnaires. Questionnaires were mailed to patients at least 5 months after admission. Responses to the question, “Do you feel foggy or unfocused 1 month after onset of COVID-19?” and “Do you feel foggy or unfocused 3 months after onset of COVID-19?” were categorized as 0 = never, 1 = rarely, 2 = occasionally, 3 = frequently, or 4 = always. This study included patients admitted to our hospital between October 10, 2020, and October 31, 2021. The participants were categorized into three groups according to their admission date: third wave, October 10, 2020, to February 28, 2021 (surveyed on May 10, 2022); fourth wave, March 1, 2021, to June 20, 2021 (surveyed on May 10, 2022); and fifth wave, June 21, 2021, to October 31, 2021 (surveyed on March 25, 2022). Questionnaires were not sent to patients who died at our hospital. Questionnaires were sent to 1061 patients, 404 of whom responded. We included 253 patients 1 month after onset of COVID-19 in the analysis, excluding those hospitalized for 1 day, who had no data on ferritin, C-reactive protein (CRP), or white blood cell counts, and those who left the Brain Fog question blank (Fig. 1). We included 229 patients when the data 3 months after the onset was used, which was fewer than 1 month as more patients left the Brain Fog question blank.

### Data collection

The following data were collected from the medical records, namely, age, sex, days in the hospital, length of hospitalization, tracheal intubation or not, peak white blood cell count, red blood cell count, platelet count, and levels of albumin, creatinine, Na, K, peak CRP, D-dimer, procalcitonin, HbA1c and treatment.

### Statistical analyses

The questionnaire was used to obtain Brain Fog scores 1 month after COVID-19 infection onset (“No symptoms”:0, “few”:1, “sometimes”:2, “frequently”:3, “always”:4). Patient characteristics and ferritin levels were compared in the presence and absence of any Brain Fog. Unless otherwise specified, categorical variables were tested using a Chi-Squared test and continuous Mann–Whitney *U* or Student’s *t* test. Furthermore, the peak ferritin level of each Brain Fog-point group was analyzed using Linear Regression.

Propensity score analyses were conducted to exclude confounders as far as possible. Some variables (Model 1; age, sex, Model 2; age, sex, CRP, and WBC, Model 3; age, sex, CRP, WBC, length of hospitalization, intubation, and maximal oxygen dose, Model 4; age, sex, CRP, WBC, length of hospitalization, intubation, maximal oxygen dose, D-dimer, creatinine, sodium, potassium, albumin, red blood cell count, platelet counts, and procalcitonin values, Model5; age, sex, CRP, WBC, length of hospitalization, intubation, maximal oxygen dose, D-dimer, creatinine, sodium, potassium, albumin, red blood cell count, platelet counts, and procalcitonin values, remdesivir, steroid therapy, antibody cocktail therapy) were regressed by Logistic Regression using the presence of Brain Fog as a predictor. Each patient propensity score was calculated as the probability of being classified into one specified group. The variables of each model were selected for the following reason, Model 1; basic social background, Model 2; inflammation-related parameters, Model 3; factors used for the determination of severity, Model 4: factors including maker of bacterial inflammation, Model 5: factors including treatment. Following greedy pair matching, each group of patients was matched using the nearest propensity scores, and we compared the two groups using the Wilcoxon signed-rank test.

Finally, the contribution of ferritin levels to the difference among patients with and without Brain Fog was tested between each COVID-19 phase using a permutation test. Specifically, the ferritin values of individuals with Brain Fog and those without Brain Fog were randomly permuted within each phase, and the median difference values of those with Brain Fog and those without brain fog were calculated based on 5000 samples. Then, a one-sided p value was calculated as the proportion of sampled values where the true median difference was located. All analyses were performed using Python 3 (Python Software Foundation, Wilmington, DE, USA).

### Ethics statement

This study was conducted according to the guidelines of the Declaration of Helsinki on Research Involving Human Subjects. All methods were performed according to the relevant guidelines and regulations and observational studies. The Ethics Committee of Higashiosaka City Medical Center approved the study design and protocol and waived the need for the requirement of Informed Consent because of the retrospective nature of the study.

## Results

### Characteristics of the patients

Among the 253 patients analyzed, 126 (49%) patients had Brain Fog 1 month after onset of COVID-19 (62 patients had category 1, 48 patients had category 2, 12 patients had category 3, and 4 patients had category 4). There were 65 patients in the third wave, 93 in the fourth wave, and 95 in the fifth wave. Days from the date of admission to questionnaire delivery were 512 ± 30 days for patients with Brain Fog and 487 ± 59 days for patients without Brain Fog in the third wave, 373 ± 24 days for patients with Brain Fog, and 386 ± 47 days for patients without Brain Fog in the fourth wave. 217 ± 25 days for patients with Brain Fog and 214 ± 29 days for patients without Brain Fog in the fifth wave; total for third-fifth waves:349 ± 120 days for patients with Brain Fog and 349 ± 119 days for patients without Brain Fog. The patients’ percentages of each wave with Brain Fog were 49%, 49%, and 51%, respectively, and there was no significant difference between with and without Brain Fog (p = 1.0, 1.0, and 0.96, respectively). Patient characteristics with and without Brain Fog are shown in Table 1. The length of hospitalization for both groups was 13.7 ± 7.0 and 12.3 ± 8.5 days, respectively. The maximum oxygen dose was higher among patients with Brain Fog than those without (5.2 ± 9.5 vs. 2.8 ± 3.0 L/min, p = 0.0069). There were 21 (16.7%) patients with tracheal intubation in the group with Brain Fog and 10 (7.9%) in the group without Brain Fog. There was no significant difference in peak CRP levels during hospitalization between patients with and without Brain Fog (7.1 ± 5.6 vs. 6.5 ± 6.0 mg/L, p = 0.41).

**Table 1:** Patient characteristics.

	With Brain Fog (n = 126)	Without Brain Fog (n = 127)	P-value
Sex, female ratio	0.48	0.46	0.85
Age, years	62 ± 16	63 ± 16	0.76
Number of patients in each phase			
Phase 3	32 (25.4%)	33 (26.0%)	1.0
Phase 4	46 (36.5%)	47 (37.0%)	1.0
Phase 5	48 (38.1%)	47 (37.0%)	0.96
Length of hospitalization, days	13.7 ± 7.0	12.3 ± 8.5	0.15
Maximal oxygen dose, L/min	5.2 ± 9.5	2.8 ± 3.0	0.0069
Intubation, number of cases	21 (16.7%)	10 (7.9%)	0.052
Laboratory data			
White blood cells, /uL (peak)	10,538 ± 4,578	10,286 ± 5,142	0.68
Red blood cells, × 10^3^/uL	464.3 ± 71.5	468.9 ± 59.7	0.28
Platelet, × 10^3^/uL	18.8 ± 7.8	19.3 ± 7.4	0.61
Albumin, g/dL	3.6 ± 0.5	3.6 ± 0.5	0.88
Creatinine, mg/dL	1.0 ± 0.8	0.9 ± 0.7	0.58
Na, mEq/L	136.5 ± 3.8	136.4 ± 3.5	0.75
K, mEq/L	3.9 ± 0.6	3.9 ± 0.5	0.98
CRP, mg/L (peak)	7.1 ± 5.6	6.5 ± 6.0	0.41
d-dimer, ug/mL	1.8 ± 3.3	1.4 ± 1.7	0.31
Procalcitonin, ng/mL	0.2 ± 0.8	0.2 ± 0.7	0.9
HbA1c, %	6.2 ± 1.2	6.4 ± 1.6	0.29
Treatment			
Remdesivir	54 (42.9%)	50 (39.4%)	0.66
Steroid therapy	102 (81.0%)	84 (66.1%)	0.01
Antibody cocktail therapy	1 (0.8%)	7 (5.5%)	0.074
Outcome			
Cure	110 (87.3%)	114 (89.8%)	0.68
Recuperation	8 (6.3%)	9 (7.1%)	1.0
Change hospital	8 (6.3%)	4 (3.1%)	0.37

Patient numbers are presented as integers and percentages. Continuous values are shown as mean ± SD. CRP for C-reactive protein.

We also analyzed the data of 229 patients based on the presence of Brain Fog 3 months after onset of COVID-19. Among the 229 patients analyzed, 95 (41%) patients had Brain Fog (46 patients had category 1, 38 patients had category 2, 7 patients had category 3, and 4 patients had category 4). Patient characteristics with and without Brain Fog are shown in Supplementary Table 1.

### Association between Brain Fog levels and ferritin levels

**Figure 2:**
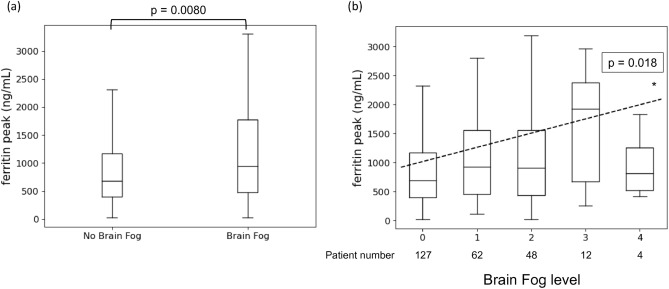
The correlation between Brain Fog levels and ferritin values. (one month after onset of COVID-19). (**a**) Mann–Whitney *U* test was evaluated. Peak ferritin levels significantly changed between the two groups (p = 0.008). (**b**) Peak ferritin levels in each Brain Fog level are expressed in a box plot. Linear Regression fitted a dashed line between ferritin peak values and Brain Fog levels (p = 0.018).

Peak ferritin levels during hospitalization were significantly higher in patients with Brain Fog than those without Brain Fog 1 month after onset of COVID-19 (median 944.3 ng/ml, interquartile range 471.9–1778.3 ng/ml vs. 685.9, 401–1168 ng/ml, p = 0.008) (Fig. 2a). Peak ferritin level was positively correlated with Brain Fog level as a continuous variable (p = 0.018) (Fig. 2b). We also analyzed the patients data 3 months after onset of COVID-19. Peak ferritin levels during hospitalization were higher in patients with Brain Fog than those without Brain Fog, but not significant (p = 0.14) (Supplementary Fig. 1A). Peak ferritin level was positively correlated with Brain Fog level as a continuous variable (p = 0.001) (Supplementary Fig. 1B).

### Propensity score analysis

The patient’s background may confound the association between ferritin levels and Brain Fog 1 month after onset of COVID-19. Hence, we developed several models using propensity scores to adjust for these factors. The background characteristics after propensity score matching are shown in Supplementary Table 2. After adjusting for confounding factors, the group with Brain Fog still had significantly higher ferritin levels than the group without Brain Fog in Models 1–4, higher but not significantly higher levels in Model 5. (Model 1: median 944.3 ng/ml, IQR 471.9–1778.25 ng/ml, vs. 687.1 ng/ml, 402.75–1179.5 ng/ml; Model 2:944.3 ng/ml, 471.9–1778.25 ng/ml, vs. 687.1 ng/ml, 402.75–1179.5 ng/ml; Model 3:831.9 ng/ml, 426.1–1431.75 ng/ml vs. 687.1 ng/ml, 392.23–1158.5 ng/ml, Model 4: 886 ng/ml, 445.58–1559.5 ng/ml, vs. 687.1 ng/ml, 391.08–1142.75 ng/ml, Model 5: 814.5 ng/ml, 417.5–1416.0 ng/ml, vs. 724.5 ng/ml, 391.65–1140.5 ng/ml) (Table 2).

**Table 2:** Logistic regression calculated propensity scores.

Variable	Patient number	Ferritin level median (IQR)	P-value
Model 1	126 vs. 126	944.3 (471.9–1778.25) vs. 687.1 (402.75–1179.5)	0.0050
Model 2	126 vs. 126	944.3 (471.9–1778.25) vs. 687.1 (402.75–1179.5)	0.020
Model 3	104 vs. 104	831.9 (426.1–1431.75) vs. 687.1 (392.23–1158.5)	0.050
Model 4	102 vs. 102	886 (445.58–1559.5) vs. 687.1 (391.08–1142.75)	0.033
Model 5	95 vs. 95	814.5 (417.5–1416.0) vs. 724.5 (391.65–1140.5)	0.25

Each parameter was included in the model (Model 1: age, sex; Model 2: age, sex, CRP, WBC; Model 3: age, sex, length of hospitalization, intubation (or not), and maximal oxygen dose, CRP, WBC; Model 4: age, sex, length of hospitalization, intubation (or not), and maximal oxygen dose, CRP, WBC, D-dimer, creatinine, sodium, potassium, albumin, RBC, platelets, procalcitonin; Model 5: age, sex, length of hospitalization, intubation (or not), and maximal oxygen dose, CRP, WBC, d-dimer, creatinine, sodium, potassium, albumin, RBC, platelets, procalcitonin, remdesivir, steroid therapy, antibody cocktail therapy). IQR interquartile ranges.

### Phase interaction

**Figure 3:**
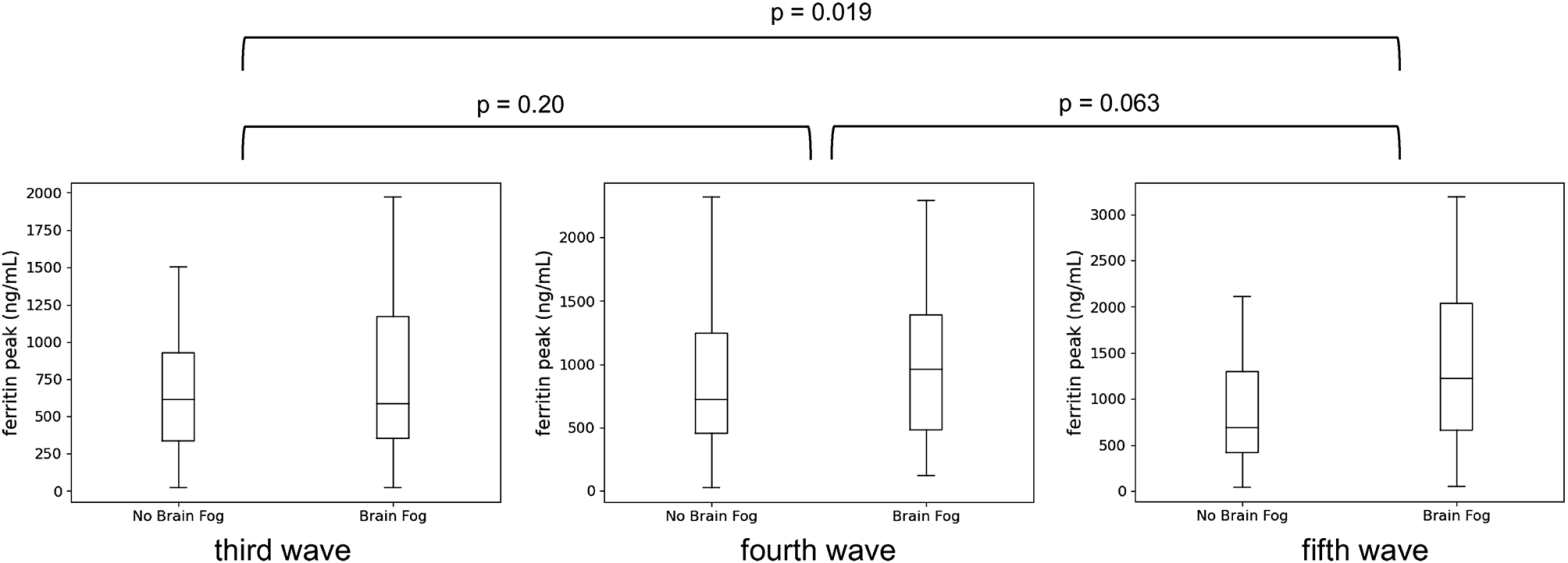
Interaction. Effect of the phases on the association between ferritin level and Brain Fog. The association between ferritin level contribution to the difference between patients with Brain Fog and without Brain Fog was evaluated in each phase using a permutation test. Significant heterogeneity was found in the association between ferritin level and Brain Fog.

This study included patients in the third-fifth wave of the COVID-19 epidemic in Japan, and the contribution of ferritin levels to the difference among patients with and without Brain Fog 1 month after onset was evaluated between each phase. The association between ferritin levels and Brain Fog was significantly stronger in the fifth wave than in the third wave (p = 0.019) (Fig. 3). The background characteristics of patients in the third-fifth wave are shown in Supplementary Table 3.

## Discussion

We investigated the symptoms of Brain Fog after COVID-19 among patients admitted to our hospital via a questionnaire. Patients with Brain Fog 1 month after onset of COVID-19 had significantly higher peak ferritin levels during hospitalization than those without, and Brain Fog levels were positively correlated with peak ferritin levels during hospitalization. After adjusting for background factors using propensity scores, ferritin levels were independently associated with Brain Fog except in Model 5. The results may provide clues to understanding the pathogenesis of Long COVID and Brain Fog, which remain unelucidated. Ferritin may have the potential as a diagnostic biomarker and may lead to the discovery of treatment methods.

Ferritin is an iron storage protein that is increased in anemia associated with chronic inflammation and is an indicator of chronic inflammation^16^. Ferritin is mainly produced in the liver and stimulated by interleukin -18 (IL-18) released from monocytes because of viral infection. Adult Still’s disease, and hemophagocytic syndrome are the representative conditions where monocytes are over-activated^17^, and serum IL-18 levels of them were reported to be high^18,19^. High serum ferritin levels also characterize them. CRP, another inflammatory response protein, is mainly produced in the liver stimulated by interleukin-1β (IL-1β) and interleukin-6 (IL-6) released from monocytes because of bacterial infection^20^. In this study, there were no significant differences in markers such as CRP and Procalcitonin, markers of bacterial infection, between patients with and without Brain Fog 1 month after onset of COVID-19. Previous research reported high serum IL-18 and IL-1β levels in Long COVID patients^4^. Another study reported that inhibition of NOD-, LRR- and pyrin domain-containing protein 3 (NLRP3) inflammasome activation, associated with innate immunity and monocyte activation, could be a therapeutic target for Long COVID^8^. Therefore, high serum ferritin levels in patients with long COVID may reflect the inflammatory properties of monocytes. Activation of monocytes and microglia and increased chemokines are observed in patients' brain meninges and choroid plexus COVID-19 infection^14,21^. Neuroinflammation, including monocytes and microglia, is also thought to be related to Brain Fog^12^. In this context, it is not surprising that serum ferritin levels can be a potential marker of neuroinflammation and Brain Fog, as shown by this study.

There are two criteria for the diagnosis of Long COVID: the CDC and the WHO definition. In the former, it begins 1 month after onset^4^, while in the latter, it continues after 3 months after onset and lasts for more than 2 months^5^. In order to utilize both criteria, we made a questionnaire which asked about symptoms 1 and 3 months after onset of COVID-19. We obtained complete Brain Fog data from 253 patients in 1 month after the onset of COVID-19, however only 229 in 3 months after the onset of COVID-19. The exact reasons why the response rate was lower regarding symptoms 3 months after onset than 1 month after onset remain unclear. The patients who left the 3 months questionnaire blank might have had some difficulties in answering the question as the patients were not able to recall their condition at that time point. Because the sample size was smaller and the data might have been influenced by unknown factors, we decided to present principal results of our analysis of the data 1 month after COVID-19 onset simply using the CDC criteria: symptoms that begin 4 weeks after onset.

The severity of COVID-19 is known to be associated with the development of Brain Fog. A previous questionnaire-based study reported patients with severe respiratory symptoms at onset and those admitted to the intensive care unit were more likely to develop Brain Fog^9^. We also showed that brain fog was associated with a higher dose of oxygen. The disease severity is a possible confounder of the association between Brain Fog and ferritin because ferritin is also associated with COVID-19 severity^22^. Therefore, using propensity scores, we showed that the relationship between Brain Fog and ferritin levels remained significant even after adjusting for background factors, including oxygen administration, one of the severity indicators of COVID-19 infection. Therefore, this indicates that ferritin is independently associated with Brain Fog. Previous studies reported that female patients are more vulnerable to long-term COVID^23^, but there was no difference by sex in this study. In Model5 which includes type of treatment, the difference in ferritin levels in patients with and without Brain Fog was not significant. Steroid therapy showed a significant difference between patients with and without Brain Fog. Corticosteroids can induce changes in cognition, mood and sleep, and can lead to changes in memory and dementia. We cannot rule out the possibility that late onset steroid psychosis may have affected the analysis and result. However, the association between ferritin levels and Brain Fog cannot be explained solely by steroid psychosis because the symptoms of steroid psychosis such as depression, mania, delirium and psychosis are different from Brain Fog symptoms and the onset of steroid psychosis is relatively acute and does not last long^24^.

The association between Brain Fog and ferritin weakened with earlier data in the third and fourth waves, while the percentage of patients with this condition remained constant in each phase. This may be due to recall bias, which can greatly affect patients' answers to a questionnaire, and the differences in patients’ backgrounds in each wave. For example, the mean age of patients in the fifth wave was younger than that of patients in the third and fourth waves. Ferritin levels are reported to be higher in older patients than in younger patients^25^. In this regard, the strength of the association between ferritin levels and Brain Fog may not be consistent across ages. In addition, the backgrounds, severity of COVID-19, and treatments for patients admitted to the hospital may not have been consistent across phases. Another possible reason is that the type of endemic viral strain is related to the onset of Brain Fog. COVID-19 can be infectious for long periods, and the intensity of inflammation induction varies from strain to strain. For instance, the δ-strain prevalent in the fifth wave^26^ caused high pathogenicity and mortality, whereas the omicron-strain, which was prevalent after the δ-strain, caused lower pathogenicity^27^. Hence, the prevalence of long-COVID and factors associated with the development of Brain Fog need to continue to be investigated.

A diagnosis of Brain Fog has not been established, and it is sometimes difficult to make a diagnosis because it can only be inferred from the patient’s complaints. However, the symptoms of Brain Fog vary, including nonspecific symptoms such as forgetfulness, cloudiness, and poor concentration^28^, and there is no gold standard for the diagnosis of Brain Fog. Some researchers used the diagnostic criteria for myalgic encephalomyelitis/chronic fatigue syndrome (ME/CFS) because the symptoms and pathophysiology are similar^29^. Patients must visit hospitals to be diagnosed with Brain Fog and undergo medical interviews, examinations, and testing specimens if required. Although the accuracy of the diagnosis is limited, questionnaires, such as the one we used in this study to collect information by phone or smartphone application, can be a convenient alternative method. One limitation of the questionnaire in this study is that whether the patient’s symptoms had existed before COVID-19 onset could not be determined, which may have affected the results. Therefore, developing biomarkers for objective diagnosis of Brain Fog is critically important. Measuring serum ferritin levels is not invasive or expensive and may be useful in identifying high-risk populations. Furthermore, it is expected to identify high-risk patients who require brain imaging such as translocator protein positron emission tomography (TSPO-PET) and magnetic resonance spectroscopy (MRS), which more accurately measures monocyte activation and are used to assess neuroinflammation observed in ME/CFS patients^30^.

One limitation of this study was that the participants might not represent the general population. Only patients admitted to a single Japanese facility were investigated. Patients that did not require hospitalization or were directly admitted to an intensive care unit were excluded. Moreover, 612 patients 1 month after COVID-19 onset did not respond to the survey. Therefore, this study included selection bias, and the generalizability of this study is limited. Another limitation was that the infectious virus strains and vaccination status of COVID-19 were unknown. The biomarkers were only evaluated during hospitalization, not from the establishment of infection to disease onset or from disease onset to hospitalization. Further research is needed to elucidate the mechanisms underlying Brain Fog after COVID-19 infection.

In conclusion, we found a positive correlation between serum ferritin levels during hospitalization and Brain Fog after COVID-19. High ferritin levels in patients with Brain Fog may reflect the contribution of chronic inflammation in the development of Brain Fog.

### Supplementary Information

Supplementary Figure 1.

Supplementary Table 1.

Supplementary Table 2.

Supplementary Table 3.

## Data Availability

The corresponding author will make available the datasets used, analyzed, or both during this study upon reasonable request.
