# Supplementary material for: Serum ferritin level during hospitalization is associated with Brain Fog after COVID-19
Source: Sci Rep. 2023 Aug 11;13:13095. doi: 10.1038/s41598-023-40011-0 (PMC10421912; doi:10.1038/s41598-023-40011-0)
Supplement: Supplementary file 1 — Supplementary Figure 1. [file 41598_2023_40011_MOESM1_ESM.pptx]

## Slide 1
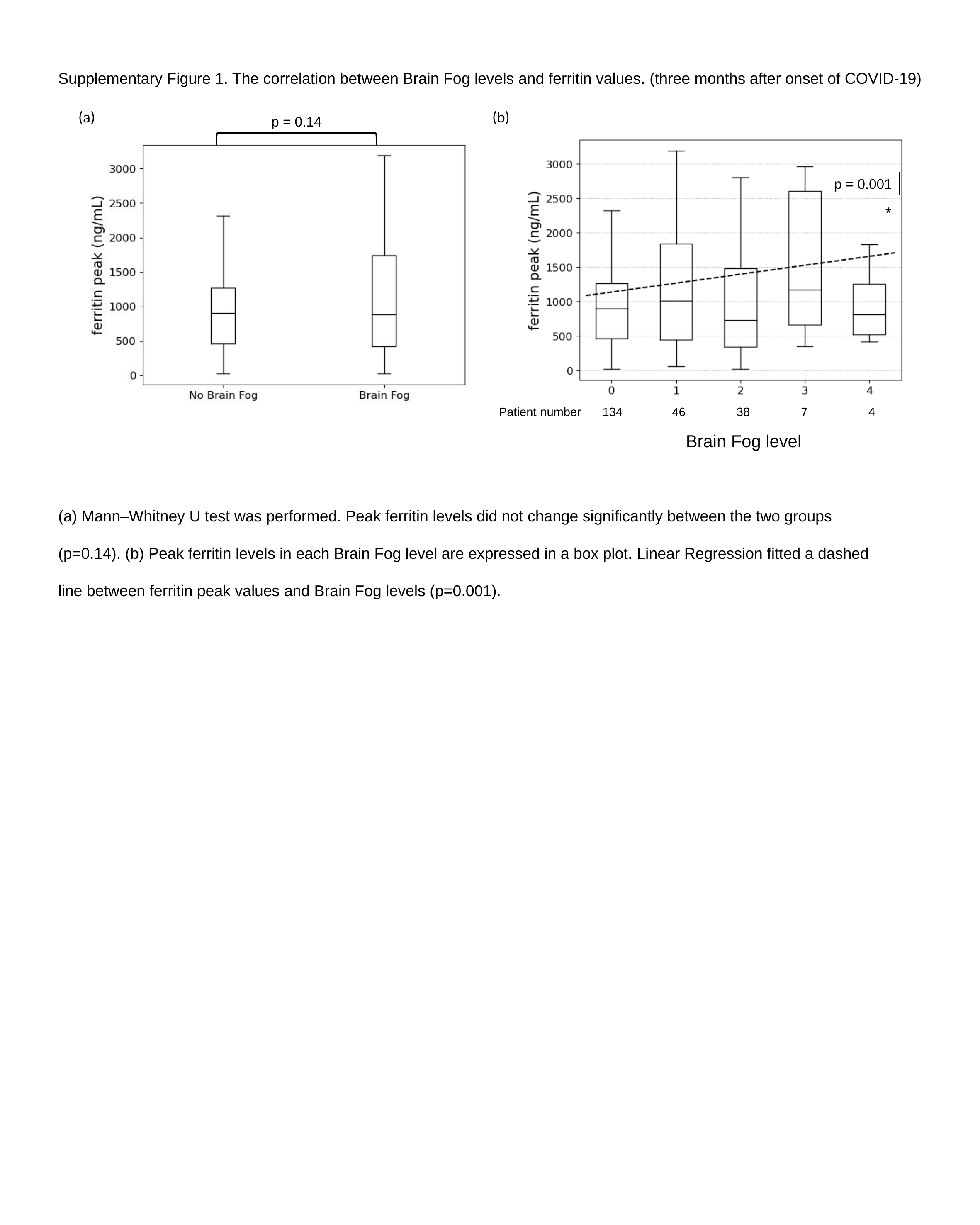

Supplementary Figure 1. The correlation between Brain Fog levels and ferritin values. (three months after onset of COVID-19)
(a)
(b)
p = 0.14
p = 0.001
*
Patient number
134
46
38
7
4
Brain Fog level
(a) Mann–Whitney U test was performed. Peak ferritin levels did not change significantly between the two groups (p=0.14). (b) Peak ferritin levels in each Brain Fog level are expressed in a box plot. Linear Regression fitted a dashed line between ferritin peak values and Brain Fog levels (p=0.001).
