# Supplementary material for: Serum ferritin level during hospitalization is associated with Brain Fog after COVID-19
Source: Sci Rep. 2023 Aug 11;13:13095. doi: 10.1038/s41598-023-40011-0 (PMC10421912; doi:10.1038/s41598-023-40011-0)
Supplement: Supplementary file 2 — Supplementary Table 1. [file 41598_2023_40011_MOESM2_ESM.docx]

Supplementary Table 1. Baseline characteristics

|  | With Brain Fog (n = 95) | Without Brain Fog (n = 134) | P-value |
| --- | --- | --- | --- |
| Sex, female ratio | 0.47 | 0.44 | 0.71 |
| Age, years | 63 ± 17 | 62 ± 16 | 0.56 |
| Number of patients in each phase | | | |
| Phase3 | 25 (26.3%) | 34 (25.3%) | 0.99 |
| Phase4 | 37 (38.9%) | 46 (34.3%) | 0.56 |
| Phase5 | 33 (34.7%) | 54 (40.2%) | 0.47 |
| Length of hospitalization, days | 13.5 ± 6.6 | 13.5 ± 9.2 | 0.97 |
| Maximal oxygen dose, L/min | 4.2 ± 4.1 | 3.2 ± 3.3 | 0.058 |
| Intubation, number of cases | 13 (13.7%) | 11 (8.2%) | 0.27 |
| Laboratory data | | | |
| White blood cells, /uL (peak) | 10,341 ± 4,470 | 10,383 ± 4,894 | 0.94 |
| Red blood cells, ×10^3^/uL | 461.2 ± 67.3 | 472.9 ± 60.5 | 0.27 |
| Platelet, ×10^3^/uL | 20.1 ± 8.4 | 18.3 ± 7.2 | 0.094 |
| Albumin, g/dL | 3.6 ± 0.5 | 3.6 ± 0.5 | 0.51 |
| Creatinine, mg/dL | 0.9 ± 0.3 | 0.9 ± 0.8 | 0.49 |
| Na, mEq/L | 136.7 ± 4.7 | 136.2 ± 3.5 | 0.38 |
| K, mEq/L | 3.9 ± 0.6 | 4.0 ± 0.6 | 0.19 |
| CRP, mg/L (peak) | 6.3 ± 5.1 | 7.2 ± 6.3 | 0.28 |
| D-dimer, ug/mL | 2.0 ± 3.8 | 1.5 ± 1.8 | 0.20 |
| Procalcitonin, ng/mL | 0.2 ± 0.9 | 0.2 ± 0.6 | 0.86 |
| HbA1c , % | 6.2 ± 1.0 | 6.4 ± 1.7 | 0.27 |
| Treatment | | | |
| Remdesivir | 41 (43.1%) | 53 (40.0%) | 0.68 |
| Steroid therapy | 74 (77.9%) | 92 (68.7%) | 0.16 |
| Antibody cocktail therapy | 0 (0.0%) | 8 (6.0%) | 0.039 |
| Outcome | | | |
| Cure | 84 (88.4%) | 116 (86.6%) | 0.83 |
| Recuperation | 4 (4.2%) | 11 (5.2%) | 0.35 |
| Change hospital | 7 (7.4%) | 7 (5.2%) | 0.70 |
| Patient numbers are presented as integers and percentages. Continuous values are shown as mean±SD. CRP for C-reactive protein. | | | |
