# Supplementary material for: Serum ferritin level during hospitalization is associated with Brain Fog after COVID-19
Source: Sci Rep. 2023 Aug 11;13:13095. doi: 10.1038/s41598-023-40011-0 (PMC10421912; doi:10.1038/s41598-023-40011-0)
Supplement: Supplementary file 3 — Supplementary Table 2. [file 41598_2023_40011_MOESM3_ESM.docx]

Supplementary Table2. Baseline characteristics after propensity score matching

|  | With Brain Fog (n=104) | Without Brain Fog (n=104) |
| --- | --- | --- |
| Sex, female ratio† | 0.49 | 0.47 |
| Age, years* | 62 ± 16 | 62 ± 15 |
| Length of hospitalization, days* | 12.7 ± 5.9 | 11.8 ± 6.9 |
| maximal oxygen dose, L/min* | 3.0 ± 3.0 | 3.0 ± 3.2 |
| Intubation, number of cases† | 9 (8.7%) | 10 (9.6%) |
| White blood cells, /uL* | 10418.8 ± 4690.7 | 9844.8 ± 4651.9 |
| Red blood cells, ×103/uL* | 465.6 ± 73.0 | 469.8 ± 58.7 |
| Platelet, ×103/uL* | 19.2 ± 8.2 | 19.2 ± 7.3 |
| Albumin, g/dL* | 3.6 ± 0.5 | 3.6 ± 0.4 |
| Creatinine, mg/dL* | 1.0 ± 0.9 | 0.9 ± 0.7 |
| Na, mEq/L* | 136.8 ± 3.8 | 136.4 ± 3.6 |
| K, mEq/L* | 4.0 ± 0.7 | 4.0 ± 0.6 |
| CRP, mg/L* | 6.6 ± 5.7 | 6.0 ± 5.3 |
| D-dimer, ug/mL* | 1.7 ± 3.5 | 1.4 ± 1.8 |
| Procalcitonin, ng/mL* | 0.2 ± 0.9 | 0.2 ± 0.7 |
| HbA1c, %* | 6.1 ± 1.3 | 6.4 ± 1.6 |

Patient numbers are presented as integers or percentages. Continuous values are shown as mean±SD. CRP for C-reactive protein.
