# Supplementary material for: Serum ferritin level during hospitalization is associated with Brain Fog after COVID-19
Source: Sci Rep. 2023 Aug 11;13:13095. doi: 10.1038/s41598-023-40011-0 (PMC10421912; doi:10.1038/s41598-023-40011-0)
Supplement: Supplementary file 4 — Supplementary Table 3. [file 41598_2023_40011_MOESM4_ESM.docx]

|  | Wave 3 (n=65) | Wave 4 (n=93) | Wave 5 (n=95) |
| --- | --- | --- | --- |
| Sex, female ratio | 0.46 | 0.51 | 0.43 |
| Age, years | 70 ± 12 | 67 ± 15 | 52 ± 14 |
| Length of hospitalization, days | 12.9 ± 7.2 | 15.1 ± 9.9 | 11.1 ± 4.8 |
| Maximal oxygen dose, L/min | 4.4 ± 12.4 | 4.5 ± 4.4 | 3.3 ± 3.3 |
| Intubation, number of cases | 15 (23.1%) | 8 (8.6%) | 8 (8.4%) |
| Laboratory data |  |  |  |
| White blood cells, /uL (peak) | 9033.7 ± 4636.7 | 11825.7 ± 5117.3 | 9970.7 ± 4425.7 |
| Red blood cells, ×10^3^/uL | 441.7 ± 67.6 | 467.4 ± 60.5 | 482.3 ± 65.5 |
| Platelet, ×10^3^/uL | 19.0 ± 7.1 | 19.9 ± 8.1 | 18.2 ± 7.5 |
| Albumin, g/dL | 3.6 ± 0.4 | 3.5 ± 0.5 | 3.6 ± 0.5 |
| Creatinine, mg/dL | 1.0 ± 1.1 | 0.9 ± 0.6 | 0.9 ± 0.6 |
| Na, mEq/L | 137.6 ± 3.7 | 136.5 ± 3.5 | 135.7 ± 3.6 |
| K, mEq/L | 4.0 ± 0.6 | 3.9 ± 0.6 | 3.9 ± 0.6 |
| CRP, mg/L (peak) | 6.4 ± 5.1 | 7.6 ± 6.0 | 6.2 ± 5.9 |
| D-dimer, ug/mL | 1.5 ± 2.2 | 1.9 ± 3.7 | 1.3 ± 1.2 |
| Procalcitonin, ng/mL | 0.2 ± 0.9 | 0.3 ± 1.0 | 0.2 ± 0.2 |
| HbA1c , % | 6.3 ± 1.4 | 6.3 ± 1.1 | 6.2 ± 1.7 |
| Ferritin, ng/ml | 784.7±644.2 | 1159.4±1218.0 | 1574.9±2301.1 |
| Treatment |  |  |  |
| Remdesivir | 2 (3.1%) | 42 (45.2%) | 60 (63.2%) |
| Steroid therapy | 37 (56.9%) | 75 (80.6%) | 74 (77.9%) |
| Antibody cocktail therapy | 0 (0%) | 0 (0%) | 9 (9.5%) |
| Outcome |  |  |  |
| Cure | 63 (96.9%) | 85 (91.4%) | 76 (80.0%) |
| Recuperation | 1 (1.5%) | 0 (0%) | 16 (16.8%) |
| Change hospital | 1 (1.5%) | 8 (8.6%) | 3 (3.2%) |

Supplementary Table3. Baseline characteristics of patients in the third-fifth wave

Patient numbers are presented as integers or percentages. Continuous values are shown as mean±SD. CRP for C-reactive protein.
